# Biology of childhood germ cell tumours, focussing on the significance of microRNAs

**DOI:** 10.1111/andr.277

**Published:** 2014-10-09

**Authors:** M J Murray, J C Nicholson, N Coleman

**Affiliations:** 1 Department of Pathology, University of CambridgeCambridge, UK; 2 Department of Paediatric Haematology and Oncology, Addenbrooke's HospitalCambridge, UK; 3 Department of Histopathology, Addenbrooke's HospitalCambridge, UK

**Keywords:** biomarker, germ cell tumour, *let-7*, *LIN28*, microRNA, miR-302/367, miR-371–373, serum

## Abstract

Genomic and protein-coding transcriptomic data have suggested that germ cell tumours (GCTs) of childhood are biologically distinct from those of adulthood. Global messenger RNA profiles segregate malignant GCTs primarily by histology, but then also by age, with numerous transcripts showing age-related differential expression. Such differences are likely to account for the heterogeneous clinico-pathological behaviour of paediatric and adult malignant GCTs. In contrast, as global microRNA signatures of human tumours reflect their developmental lineage, we hypothesized that microRNA profiles would identify common biological abnormalities in all malignant GCTs owing to their presumed shared origin from primordial germ cells. MicroRNAs are short, non-protein-coding RNAs that regulate gene expression via translational repression and/or mRNA degradation. We showed that all malignant GCTs over-express the miR-371–373 and miR-302/367 clusters, regardless of patient age, histological subtype or anatomical tumour site. Furthermore, bioinformatic approaches and subsequent Gene Ontology analysis revealed that these two over-expressed microRNAs clusters co-ordinately down-regulated genes involved in biologically significant pathways in malignant GCTs. The translational potential of this finding has been demonstrated with the detection of elevated serum levels of miR-371–373 and miR-302/367 microRNAs at the time of malignant GCT diagnosis, with levels falling after treatment. The tumour-suppressor *let-7* microRNA family has also been shown to be universally down-regulated in malignant GCTs, because of abundant expression of the regulatory gene *LIN28*. Low *let-7* levels resulted in up-regulation of oncogenes including *MYCN*, *AURKB* and *LIN28* itself, the latter through a direct feedback mechanism. Targeting *LIN28*, or restoring *let-7* levels, both led to effective inhibition of this pathway. In summary, paediatric malignant GCTs show biological differences from their adult counterparts at a genomic and protein-coding transcriptome level, whereas they both display very similar microRNA expression profiles. These similarities and differences may be exploited for diagnostic and/or therapeutic purposes.

## Aims

The aim of this review was to focus on the biological studies of malignant germ cell tumours (GCTs) and highlight similarities and differences between tumours arising during childhood and those arising in adulthood. In addition to considering genomic and protein-coding transcriptomic changes, we will emphasize findings from expression profiling studies of short non-protein-coding RNAs, termed microRNAs, in GCTs. Our improving knowledge of the molecular mechanisms underlying the pathogenesis of GCTs is contributing to the identification of new biomarkers and therapeutic targets, and the development of clinico-biological algorithms for disease segmentation and risk stratification. Combined with the developing collaborations between international clinical trial groups, we can be cautiously optimistic that these approaches will, in the foreseeable future, improve the clinical management of the children, adolescents and young adults affected by this disease (Murray *et al*., 2009; Collinson *et al*., 2014; Stoneham *et al*., 2014).

## Background

Germ cell tumours are clinically and pathologically complex neoplasms that occur from the neonatal period through to late adulthood (Murray & Nicholson, 2010). They show extensive clinico-pathological heterogeneity, for example in their incidence rate, presentation and histology. Only 50% of GCTs present at gonadal sites during childhood, whereas this figure increases to 95% in adulthood (Murray & Nicholson, 2010). GCTs display a characteristic bimodal age distribution in childhood, with a relatively high incidence rate in the first few years of life, which then declines to very low levels around 5 years of age, before increasing again in adolescence. As a result, their incidence in the paediatric (four per million) and older adult (14 per million) populations remains markedly lower than in teenagers and young adults (TYAs) (60 per million in males and eight per million in females) (Murray *et al*., 2009).

Despite these clinical differences, all GCTs are believed to originate from primordial germ cells (PGCs), regardless of patient age, tumour site and histological subtype (Teilum, 1965). Despite the common origin theory, the histological appearance of GCTs varies depending on the type and degree of subsequent differentiation. Those tumours that show extensive somatic differentiation are referred to as teratomas and are generally considered benign, particularly in paediatric practice. Malignant GCTs show varying degrees of differentiation and are classified into seminoma (undifferentiated) and non-seminomatous tumours [yolk sac tumour (YST), embryonal carcinoma (EC) and choriocarcinoma (CHC)]. The latter tumours show yolk sac, embryonal and trophoblastic/placental-like differentiation respectively (Murray & Nicholson, 2010). In this review, the term seminoma is used to collectively describe testicular seminomas, ovarian dysgerminomas and extragonadal germinomas. Mixed malignant GCTs also occur, which are composed of more than one histological subtype. Clinical differences between GCTs arising in children and adults, along with experimental data, such as the maturational stage and imprinting status of the originating PGC, led Oosterhuis and Looijenga to propose a classification system where GCTs are divided into five types (Oosterhuis & Looijenga, 2005). Relevant to this review, ‘Type I’ tumours occur in neonates and infants <5 years of age and predominantly comprise non-seminomatous teratomas and YSTs. ‘Type II’ tumours include both non-seminomatous and seminomatous GCTs and include testicular disease in patients >15 years and ovarian tumours in those aged >4 years (Oosterhuis & Looijenga, 2005).

## Clinical perspective

Malignant GCTs rapidly became a highly curable disease, even when diagnosed at advanced clinical stages, with the advent of cisplatin-based chemotherapy in the 1970s (Einhorn & Donohue, 1977; Williams *et al*., 1987). However, up to 20% of patients may still eventually die of their disease. Although paediatric GCTs were initially treated with ‘adult’ type cisplatin-based regimes, the long-term sequelae of such schedules were substantial, including pulmonary fibrosis (Osanto *et al*., 1992), nephrotoxicity (Bosl *et al*., 1986), neuropathy (Glendenning *et al*., 2010) and ototoxicity (Bokemeyer *et al*., 1996a; Strumberg *et al*., 2002). Of particular concern are reports of increased risks of early onset cardiovascular disease (Huddart *et al*., 2003) and second malignancies (Travis *et al*., 1997, 2005) in survivors of malignant GCT disease. These side effect profiles resulted in the adoption of a carboplatin-based approach instead for affected children in the UK, with comparable survival for all tumour stages (Mann *et al*., 2000). However, carboplatin therapy has been shown to be inferior to cisplatin in adults with malignant testicular GCTs (TGCTs), although lower dose carboplatin regimes have typically been used in this age-group owing to the increased risk of myelosuppression (Bokemeyer *et al*., 1996b1996b; Horwich *et al*., 1997; Collinson *et al*., 2014). When attempting to identify the optimal treatment schedules for such patients however, the currently available clinical risk stratification systems are not sophisticated enough to truly distinguish those GCT patients who will have excellent outcomes from those destined to have poor outcomes. For example, within the International Germ Cell Consensus Classification (IGCCC) (1997) high-risk group it is not possible to identify upfront at the time of diagnosis the 55% of patients who will be chemotherapy resistant (and have either refractory disease or show subsequent relapse), from the 45% for whom four courses of standard cisplatin-based chemotherapy (BEP) will be sufficient for cure. Consequently, we need to extend our knowledge of GCT biology and use it to develop combined clinico-biological risk stratification algorithms, improved biomarkers and targets for the development of novel therapeutic agents for patients with malignant GCTs. This should ultimately lead to improved cure rates for those patients who currently have poor outcomes in the IGCCC (or equivalent risk stratification) intermediate- and high-risk disease groups and allow reduction of toxicity in those patient groups truly defined as low-risk.

## Genomic changes

Atkin & Baker (1982) first described the detection of an isochromosome of the short arm of chromosome 12 [i(12p)] in TGCTs over three decades ago. This abnormality occurs in ∽80% of adult TGCTs, whereas more limited gain of 12p genomic material is almost invariably present in the remaining cases (Collinson *et al*., 2014). 12p gain is also a common cytogenetic event in ovarian GCTs (Kraggerud *et al*., 2000). Gain of 12p occurs regardless of histological subtype in adult gonadal malignant GCTs. In contrast, 12p gain was initially thought to occur rarely in paediatric malignant GCTs (Collinson *et al*., 2014), for example described in only 1/16 (6%) of cases in a small cohort of gonadal and extragonadal tumours that exclusively comprised YST histology (Perlman *et al*., 2000). A larger study of 33 paediatric gonadal and extragonadal malignant GCTs identified 12p gain in just three cases (8%; one testicular and two ovarian cases) (Schneider *et al*., 2001). Bussey *et al*. (2001) identified 12p gain in 6/53 (11%) paediatric GCT cases and identified increased prevalence in male (28%) compared with female (3%) patients. More recently, a paediatric study which included a large proportion of ovarian malignant GCTs and both seminoma and YST histology, revealed 12p gain in 44% of cases, with an increasing incidence with age: 53% in those aged 5–16 years compared with 29% in those <5 years (Palmer *et al*., 2007). These findings suggest that genomic copy number imbalances can distinguish GCT subgroups primarily by patient age, rather than by tumour site or histology.

As the only consistent structural chromosomal abnormality in malignant GCTs of adult patients, 12p gain is implicated in invasive malignant GCT development. The observation that the pre-invasive testicular lesion intratubular germ cell neoplasia unclassified demonstrates a similar pattern of overall genomic changes to those seen in invasive TGCTs, except for 12p gain, supports this theory (Rosenberg *et al*., 2000; Summersgill *et al*., 2001; Ottesen *et al*., 2003). Reports suggest that 12p gain in TGCTs results in the activation of key stem cell genes which promote cellular proliferation (Korkola *et al*., 2006). However, the identification of which specific genes on 12p are the fundamental universal ‘drivers’ of malignant GCT pathogenesis and which are merely ‘passengers’ has been difficult to achieve. For example, adult testicular seminomas and ECs over-express stem cell genes (e.g. *NANOG*) located at the common region of 12p gain (12p13.31), suggesting that they are likely to be important in tumorigenesis. However, other non-seminomatous tumours, for example YSTs, which also have 12p gain, do not over-express these genes, suggesting other mechanisms must be important.

Other genomic copy number imbalances in malignant GCTs have also been described in adults. Commonly observed imbalances include gains on chromosomes 1, 7, 8, 12, 21 and X, as well as losses on 4, 5, 11, 13 and 18. However, none of these reported abnormalities are as consistent as gain of 12p (Collinson *et al*., 2014). A wide range of copy number imbalances has also been described in paediatric malignant tumours, including gains on chromosomes 1q, 2p, 3, 7, 8, 13, 14, 20q, 21 and X, as well as losses on 1p36, 4q, 6q, 11, 13 and 18 (Collinson *et al*., 2014).

In the last few years, high-resolution approaches have become available to interrogate genomic abnormalities and regions of interest in far greater detail than has previously been possible. A small study of 25 adult malignant testicular seminomas has been published using this technique, which re-confirmed the presence of 12p gain (LeBron *et al*., 2011). Comparison of early- and late-stage disease identified copy number variations that correlated with progression, including 4q, 5p, 9q, 13q and 20p deletions and 9q and 13q amplifications (LeBron *et al*., 2011). Similar studies in childhood GCTs are ongoing and are likely to reveal many novel aberrations which may account for the observed differences in clinical behaviour and outcomes of these tumours.

## Protein-coding gene expression

**Figure 1:**
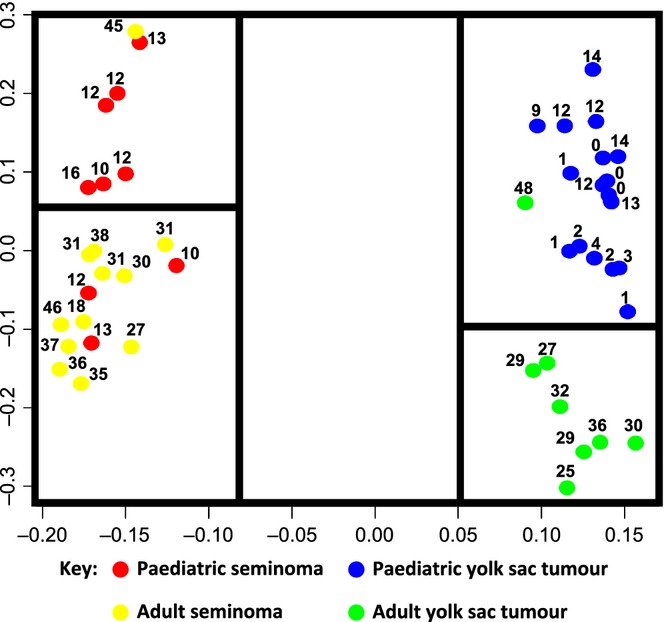
Principal component analysis of paediatric and adult malignant germ cell tumour (GCT) protein-coding gene expression data [adapted from (Palmer *et al*., 2008)]. Each malignant GCT sample is represented by a coloured circle; the number next to the circle shows the age of the relevant patient in years. The distance between samples represents biological (protein-coding transcriptome) differences. Consequently, those samples clustering close together demonstrate biological similarity, whereas those that are segregated are more dissimilar.

Protein-coding transcriptomic studies have predominantly been performed in adult TGCTs. A recent review article of 23 such studies highlighted common gene changes in TGCTs, elucidating transcriptional changes associated with malignant transformation and with differentiation patterns of malignant GCTs (Alagaratnam *et al*., 2011). This study implicated both known (*KRAS*, *MYCN* and *TPD52*) and novel (*CCT6A*, *IGFBP3* and *SALL2*) cancer genes in TGCT pathogenesis. Gene expression patterns in malignant GCTs characteristic of embryonic stem cells (ESCs) were confirmed and a distinctive transcriptomic programme was identified for individual histological subtypes (Alagaratnam *et al*., 2011). For seminoma, this included *LZTS1*; for EC, *DNMT3B*, *GAL* and *GPC4*; for CHC *CGA*; and for YSTs *AFP*, *APOA2*, *BMP2*, *VTN* and *OTX2* (Alagaratnam *et al*., 2011). Similarly, global mRNA gene expression profiles in paediatric malignant GCTs completely segregated the two main histological subtypes, YSTs and seminomas (Palmer *et al*., 2008). Within individual histological subtypes, however, tumours shared similar protein-coding transcriptomes, regardless of whether they were gonadal or extragonadal in origin (Palmer *et al*., 2008). As seen for adult seminomas (Korkola *et al*., 2006), paediatric seminomas were enriched for genes associated with pluripotency and the undifferentiated state [e.g. *NANOG*, *POU5F1* (*OCT3/4*), *TFAP2C* and *UTF*] and paediatric YSTs were associated with genes such as *AFP*, those involved in differentiation (*KRT8*, *KRT19*), lipid metabolism (*APOA1*, *APOA2*) and proliferation pathways (Palmer *et al*., 2008). Interestingly, global mRNA profiles also segregated paediatric cases from adult malignant TGCTs of the same tumour subtype, suggesting that for mRNA expression, histological subtype is the main discriminator, and then patient age (Palmer *et al*., 2008) (Fig.1). Alterations in hormonal status that accompany puberty may partly account for the observed differences in mRNA expression profiles between paediatric and adult GCTs. Subsequent to these studies, a prognostic mRNA gene expression signature predictive of overall survival was identified and validated in adult males with non-seminomatous malignant GCTs (Korkola *et al*., 2009). Future studies of GCTs affecting patients in both childhood and adolescence will need to assess whether such an ‘adult’ mRNA signature is prognostic in these younger age-groups. If not, it will be important to identify which specific biomarkers are predictive of clinical outcome in paediatric and adolescent GCTs, to improve risk stratification and management.

During the last decade, key insights into the molecular basis of cancer have been elucidated, resulting in a growing understanding of the complex cancer-associated signalling pathways that underlie tumour formation and progression. Among these pathways, *Wnt*, *TGF-beta/BMP*, *PI3K/AKT/mTOR*, *RAS/RAF* and *VEGF* signalling are of special interest, as they have driven the development of a new generation of anti-cancer drugs which target specific molecular events/mutations (Murray & Schönberger, 2014). Importantly, these pathways have also been identified as dysregulated in malignant GCTs, suggesting possible novel therapeutic targets (Murray & Schönberger, 2014). The generally more aggressive clinical behaviour of adult malignant GCTs in comparison with their paediatric counterparts is likely to be due, at least in part, to the differential expression of individual protein-coding genes within those pathways (Palmer *et al*., 2008). This differential gene expression may be owing to a number of factors, including genomic copy number alterations (Palmer *et al*., 2007) or differences in epigenetic mechanisms observed between the two age-groups of patients.

Differential expression of genes involved in cancer-associated signalling pathways has been shown between the two main pure histological subtypes of childhood malignant GCTs, namely YSTs and seminomas. An mRNA microarray analysis identified significant differential expression of genes involved in the *Wnt* pathway between these two subtypes (Fritsch *et al*., 2006). Besides *WNT13*, *beta-catenin* was also differentially expressed and showed nuclear translocation in over 50% of YSTs, in contrast to seminomas, where this occurred rarely. These differences suggested activation of *Wnt* signalling in YSTs. Subsequent gene expression analysis of intra- and extracellular regulators of the WNT pathway confirmed the differential expression of several genes between the two subtypes, for example *SFRP2* and *DKK1*, predominantly owing to epigenetic mechanisms (Schönberger *et al*., 2010). Methylation of the DNA of these genes is therefore likely to account for the over-expression of *Wnt/beta-catenin* pathway genes seen in YSTs (Fritsch *et al*., 2006; Palmer *et al*., 2008). Similarly, differential protein-coding gene expression leads to activation of the *TGF-beta/BMP* pathway in YSTs, in contrast to undifferentiated tumours such as seminomas, where *BMP* pathway activity is absent (Fustino *et al*., 2011).

*KIT* and its ligand *KITLG* (steel factor) are not only involved in normal PGC development, resulting in oogenesis and spermatogenesis, but are also implicated in GCT development (Gilbert *et al*., 2011). They are known to activate the *PI3K/AKT/mTOR* and *RAS/RAF* pathway, and consequently, recent research has focused on *KIT/KITLG* in GCTs. Several immunohistochemical studies of adult GCTs detected KIT in seminomas in contrast to non-seminomatous tumours such as YSTs (Bokemeyer *et al*., 1996c1996c; Kemmer *et al*., 2004; Nakai *et al*., 2005; Biermann *et al*., 2007; Nikolaou *et al*., 2007). In addition, mutations in *KIT* in codon 816 (exon 17) are associated with the development of bilateral GCTs (Looijenga *et al*., 2003) and advanced stages of ovarian dysgerminoma (Cheng *et al*., 2011). Genetic and protein analysis identified different gain-of-function mutations in the *KIT* gene (D816V, D816H) in seminomas, resulting in phosphorylation of KIT and PI3K and therefore constitutive activation of the *PI3K* pathway, even in the absence of KITLG (Nakai *et al*., 2005). Unfortunately, although seminomas may harbour activating *KIT* mutations, responses in clinical studies of the KIT tyrosine kinase inhibitor imatinib mesylate have been disappointing, with no complete or even partial remissions (Einhorn *et al*., 2006). However, this is not entirely surprising as only those patients with mutations in exon 11 of KIT tend to respond to imatinib therapy. In vitro studies suggest that alternative tyrosine kinase inhibitors, such as dasatinib, which target exon 17 mutations, may be more promising as treatment options in vivo (Schittenhelm *et al*., 2006).

In many human cancers, mutations in the *KRAS* or *BRAF* gene lead to activation of the *RAS/RAF* pathway, resulting in over-expression of its target *MAPK1* (*ERK*). Although *MAPK1* is globally expressed in adult GCTs, mutations of *KRAS* and *BRAF* are rare events, pointing to the involvement of *KIT* as an upstream protein in activation of *RAS*/*RAF* signalling (McIntyre *et al*., 2005; Sommerer *et al*., 2005). Nevertheless, a European study identified that the *BRAF* mutation V600E is detectable in a subgroup of chemotherapy-resistant adult GCTs and correlates significantly with the presence of microsatellite instability (Honecker *et al*., 2009). Of note, however, this finding could not be verified in a recent US study of adult patients with malignant GCTs (Feldman *et al*., 2014), nor in a large cohort of paediatric GCTs (Masque-Soler *et al*., 2012).

## Non-protein-coding gene (microRNA) expression

The identification of molecular abnormalities that are shared across the diverse spectrum of malignant GCTs is particularly important, as these are likely to be of fundamental significance in disease pathogenesis. MicroRNAs are short, non-protein-coding RNAs that regulate the expression of protein-coding genes through interactions with binding sites in the 3′ untranslated region (3′UTR) of target mRNAs, and whose expression profiles are dysregulated in cancer (Palmer *et al*., 2010). MicroRNAs may act directly as oncogenes or tumour suppressor genes through their interactions with mRNA targets (Palmer *et al*., 2010). As microRNA profiles reflect the developmental lineage of tumours (Lu *et al*., 2005), it was speculated that undertaking microRNA expression analysis in GCTs might identify such shared molecular abnormalities (Palmer *et al*., 2010).

### Over-expressed tissue microRNAs

An early study revealed that the miR-371–373 microRNA cluster was highly expressed in adult TGCTs (Voorhoeve *et al*., 2006), suggesting that it may act as a potential novel oncogene in TGCTs via inhibition of the tumour suppressor gene *LATS2* (Voorhoeve *et al*., 2006). Subsequently, it was confirmed that the miR-371–373 cluster was over-expressed in adult gonadal malignant GCTs, when compared with normal testis controls (Gillis *et al*., 2007).

**Figure 2:**
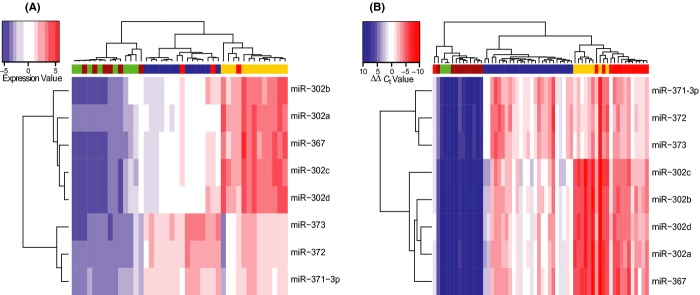
Differential expression of the miR-371–373 and miR-302/367 clusters in malignant germ cell tumours (GCTs) [adapted from (Palmer *et al*., 2010)]. Hierarchical clustering analysis based on the eight main microRNAs from the miR-371–373 and miR-302/367 clusters (rows) segregates (A) paediatric and (B) adult malignant GCT samples from non-malignant controls (comprising benign teratomas and normal gonadal controls) (columns). In the heatmap, red represents relative microRNA over-expression and blue represents under-expression. Green columns = normal gonadal controls; brown = teratoma; blue = seminoma; yellow = yolk sac tumour; red = embryonal carcinoma.

Next, a global microRNA profiling study (*n* = 615 microRNAs) reported data from 48 paediatric samples, including gonadal and extragonadal (including intracranial) malignant GCTs (Palmer *et al*., 2010), and compared the profiles obtained with those from the adult gonadal GCT study described above (Gillis *et al*., 2007). The majority of differentially expressed microRNAs in paediatric GCTs were down-regulated (Palmer *et al*., 2010), consistent with observations in other malignancies (Lu *et al*., 2005). However, the most significant finding was that the miR-371–373 and miR-302/367 clusters were over-expressed in all malignant GCTs, independent of patient age (paediatric or adult), tumour histological subtype (YST, seminoma or EC) or anatomical site (gonadal or extragonadal) (Palmer *et al*., 2010). This finding was the first common biological abnormality identified in all malignant GCTs and was consistent with the presumed common origin of GCTs from PGCs (Palmer *et al*., 2010). Importantly for potential clinical use as highly sensitive and specific universal biomarkers of malignant GCTs, the expression levels of the eight main microRNA members from the miR-371–373 and miR-302/367 clusters accurately segregated malignant GCTs from the non-malignant group, comprising fetal and gonadal control samples and benign teratomas (Fig.2) (Palmer *et al*., 2010).

As both the miR-371–373 and miR-302/367 microRNA clusters are ESC-specific pluripotency markers (Suh *et al*., 2004; Thomson *et al*., 2006; Lakshmipathy *et al*., 2007; Barroso-del Jesus *et al*., 2008; Laurent, 2008), the co-ordinate expression of microRNAs from these clusters in malignant GCTs either represents the persistence of an embryonic pattern of microRNA expression that is not present in normal, somatically differentiated tissues, or acquired re-expression, regulated by an as yet undetermined mechanism(s) (Palmer *et al*., 2010). Of potential functional significance, six of the eight main microRNAs from the miR-371–373 clusters were noted to contain an identical ‘seed’ region at 5′ nucleotide positions 2–7 (2–7nt), critical for determining binding specificity to mRNA targets (Palmer *et al*., 2010). In addition to the universal miR-371–373 and miR-302/367 over-expression findings, each subtype of malignant GCT was additionally characterized by specific abnormalities of microRNA expression (Palmer *et al*., 2010). Interestingly, however, the striking differences in mRNA expression that were previously observed between paediatric and adult malignant GCTs (Palmer *et al*., 2008) were not reflected by similar differences in microRNA expression profiles (Palmer *et al*., 2010).

As microRNAs regulate gene expression via translational repression and mRNA destabilization (Gautier *et al*., 2004; Esquela-Kerscher & Slack, 2006; Giraldez *et al*., 2006), the latter resulting in mRNA expression changes (Lim *et al*., 2005; Calin & Croce, 2006), matched global mRNA profiles were ranked by expression change and then assessed by the Sylamer bioinformatic algorithm (van Dongen *et al*., 2008). This analysis showed that the identical seed region shared by microRNAs from the over-expressed miR-371–373 and miR-302/367 clusters resulted in a global down-regulation of target mRNAs in malignant GCTs and was therefore of functional significance (Palmer *et al*., 2010). Using Gene Ontology analysis, it was demonstrated that these mRNA targets mediated important cancer-associated cellular processes, such as signal transduction, cell cycle, development and morphogenesis (Palmer *et al*., 2010). Interestingly, the same microRNAs have been shown to be essential for regulating G1-S transition and promoting rapid proliferation in ESCs (Wang *et al*., 2008; Wang & Blelloch, 2009).

The profiles of the differentially expressed microRNAs that segregated the two main pure histological subtypes of malignant GCT, YST and seminoma, were very similar in both adult and paediatric patients, and may account for the observed differences in clinical outcome between the two tumour types (Murray *et al*., 2010). In particular, further over-expression of the miR-302/367 cluster was observed in YSTs compared with seminomas, which resulted in the down-regulation of cancer-associated protein-coding genes (Murray *et al*., 2010). Furthermore, in another study the relative miR-302/367 over-expression in YSTs was associated with increased bone morphogenetic protein (BMP) signalling activity in YSTs (compared with seminomas), presumably via multiple predicted mRNA targets in the transforming growth factor*–*beta/BMP pathway (Fustino *et al*., 2011).

Of note, the miR-371–373 cluster is involved in maintaining the pluripotent state in ESCs and germline stem cells, whereas miR-302/367 members are induced during the first stages of differentiation (Zovoilis *et al*., 2008). As miR-302/367 expression is lost in cells and tissues showing somatic differentiation (Suh *et al*., 2004; Barroso-del Jesus *et al*., 2008), it is likely that levels peak during early extraembryonic differentiation. If so, dynamic changes in miR-302/367 levels in normal embryonic development (Stadler *et al*., 2010) would be mirrored in GCTs showing equivalent differentiation states (Murray *et al*., 2010). Those GCTs with extraembryonic differentiation (i.e. YSTs and potentially CHC) would display high levels compared with undifferentiated tumours (seminomas), with a reduction to virtually undetectable levels in somatically differentiated tumours (teratomas) (Murray *et al*., 2010), in which microRNA profiles are almost identical to normal gonadal tissues (Palmer *et al*., 2010). Another study of childhood GCTs demonstrated DNA hypermethylation in YSTs compared with seminomas, coincident with higher levels of expression of the DNA methyltransferase, *DNMT3B* (Jeyapalan *et al*., 2011). Of particular interest, *DNMT3B* is a predicted target of the miR-29 family (miR-29a, -29b and -29c). Consistent with these observations, the miR-29 family is under-expressed in YSTs, when compared with seminomas (Palmer *et al*., 2010), and may account for clinical differences in behaviour.

In summary, miR-371–373 and miR-302/267 cluster over-expression occurs in all malignant GCTs, regardless of patient age, histological subtype and anatomical site of disease (Palmer *et al*., 2010). This is in contrast to mRNA profiles in malignant GCTs, where no such universal findings were described (Palmer *et al*., 2008). However, similar to mRNA profiles (Alagaratnam *et al*., 2011), microRNA expression differences between each individual histological subtype vs. control samples were identified, in addition to the key over-expressed miR-371–373 and miR-302/367 clusters (Palmer *et al*., 2010). Another similarity to protein-coding gene profiles are that there are microRNA expression differences between histological subtypes, for example seminoma and YST (Murray *et al*., 2010). Discordant with the findings for mRNA analysis (Fig.1), there do not appear to be any major microRNA expression differences within histological subtypes by patient age (i.e. in paediatric vs. adult samples) (Palmer *et al*., 2010).

### Detection of serum microRNAs

Although the serum biomarkers alpha-fetoprotein (AFP) and human choriogonadotrophin (HCG) assist malignant GCT diagnosis, they have limitations in sensitivity and specificity (Murray & Nicholson, 2011). AFP is produced by YST components and HCG predominantly by CHC; consequently neither marker is raised in all cases of malignant GCT and both show elevations in non-malignant conditions (Murray & Nicholson, 2011). A biomarker that offered greater sensitivity and specificity for diagnosing or monitoring malignant GCTs would therefore be of considerable clinical value (Murray *et al*., 2011).

There are no reports demonstrating the clinical utility of serum levels of protein-coding mRNAs in malignant GCTs. Moreover, potential mRNA biomarkers can be subject to considerable variation in levels, for technical as well as biological reasons. In particular, mRNAs are inherently unstable at room temperature and rapidly degrade in blood-based samples that are not stored correctly (Rainen *et al*., 2002; Viprey *et al*., 2007). Furthermore, a small study describing non-protein-coding *XIST* transcripts in the plasma of male patients with TGCTs (Kawakami *et al*., 2004) had low sensitivity and has not been confirmed by others.

In contrast, microRNAs released from tumour cells into the bloodstream are stable because of protection from RNAse degradation by packaging within membrane-bound exosome particles (Caby *et al*., 2005; Valadi *et al*., 2007). MicroRNAs show stability in serum samples subjected to multiple freeze–thaw cycles (Chen *et al*., 2008; Mitchell *et al*., 2008) and in those left at room temperature prior to processing (Mitchell *et al*., 2008). Furthermore, a good correlation exists between levels of individual microRNAs in serum and plasma samples obtained from the same patient (Mitchell *et al*., 2008). As a result, blood-based microRNAs show considerable promise for cancer diagnosis and monitoring (Murray *et al*., 2011).

**Figure 3:**
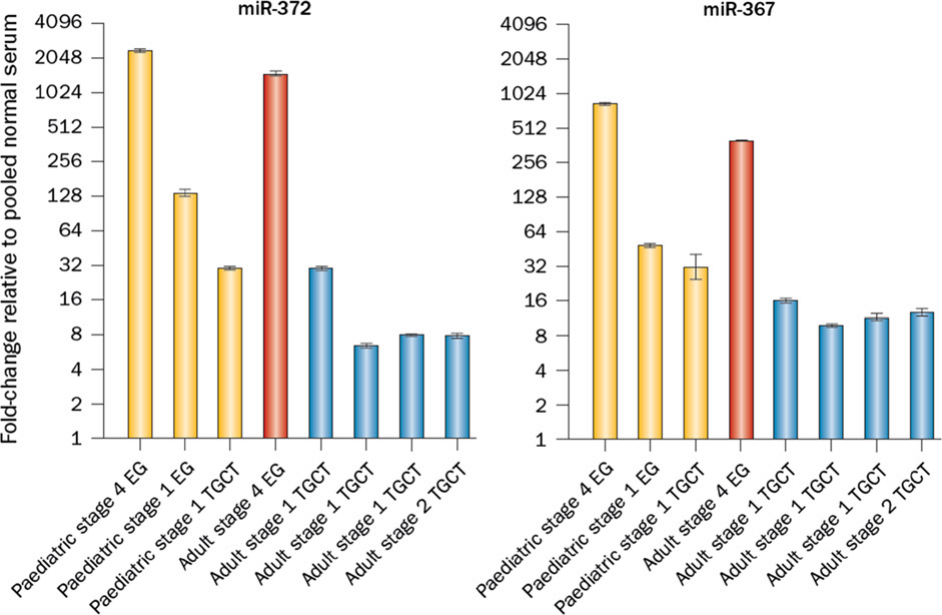
MicroRNAs from the miR-371–373 and miR-302/367 clusters as novel serum biomarkers of malignant germ cell tumours (GCTs) [adapted from (Murray & Coleman, 2012)]. Levels of miR-372 from the miR-371–373 cluster (left) and miR-367 from the miR-302/367 cluster (right) in the serum at the time of diagnosis in eight malignant GCTs of different patient age, anatomical site and histological subtype. Key: EG, extragonadal; TGCT, testicular germ cell tumour. Yellow columns = yolk sac tumour samples; red = embryonal carcinoma; blue = seminoma.

The first report of serum microRNA expression in malignant GCTs contained a detailed multiplexed qRT-PCR methodology and demonstrated elevated serum levels of all eight main members of the miR-371–373 and miR-302/367 clusters in the serum of a paediatric patient compared with pooled normal serum (Murray *et al*., 2011). Levels of miR-372 were over 700-fold higher at malignant GCT diagnosis and fell to normal levels during treatment and in uneventful clinical follow-up (Murray *et al*., 2011). Further study of malignant GCTs, including TGCTs, across a range of representative ages (paediatric and adult), anatomical sites (gonadal and extragonadal) and histological subtypes (YST, seminoma and EC) confirmed universal over-expression at diagnosis of serum levels of miR-372 and miR-367 (Fig.3) (Murray & Coleman, 2012). Importantly, the majority of the cases described were marker-negative by serum AFP and HCG estimation. There was a potential association between serum levels of both miR-372 and miR-367 and total tumour volume at diagnosis (Murray & Coleman, 2012).

Using the same qRT-PCR methodology (Murray *et al*., 2011; Murray & Coleman, 2012), these initial findings have been extended. Elevated levels of the three main microRNAs from the miR-371–373 cluster were demonstrated in stage 1 TGCT patients at the time of diagnosis compared with control serum, and fell to normal levels within 5 days of orchidectomy (Belge *et al*., 2012; Dieckmann *et al*., 2012). The majority of patients described also had ‘marker-negative’ disease by serum AFP/HCG quantification (Belge *et al*., 2012; Dieckmann *et al*., 2012). In these studies, the highest serum levels were noted for miR-371a-3p, which also fell the most dramatically following treatment (Belge *et al*., 2012; Dieckmann *et al*., 2012). In addition, in advanced stage cases, serum levels of miR-371–373 microRNAs only reduced to the normal range after treatment with surgery and chemotherapy (Dieckmann *et al*., 2012). Importantly, for the benign teratoma cases included in the study, serum miR-371–373 levels at the time of diagnosis were in the range of the controls, demonstrating the potential clinical utility of these serum microRNAs to distinguish malignant disease from teratoma, for which the management approaches may be very different (Dieckmann *et al*., 2012).

Subsequently, a large collaborative serum microRNA qRT-PCR study employed a pipeline using non-human spike-in RNAs to control for technical variation in sample preparation (Gillis *et al*., 2013). Across all the datasets studied, a consistent, highly significant increase in the levels of four microRNAs (miR-371a-3p, miR-372, miR-373 and miR-367) was observed in serum from all patients with malignant GCTs at the time of diagnosis. Area under the curve values in receiver operator characteristic analyses were 0.88 (miR-371a-3p), 0.91 (miR-372), 0.96 (miR-373) and 0.94 (miR-367) (Gillis *et al*., 2013), significantly outperforming AFP and HCG. Combined use of these four microRNAs with AFP/HCG identified all diagnostic malignant GCT samples correctly. The sensitivity of each marker was between 80 and 90% at a specificity cut-off of 90% (Gillis *et al*., 2013).

Thus, microRNAs of the miR-371–373 and miR-302/367 clusters are emerging as promising bodyfluid biomarkers to improve clinical management of malignant GCTs. A further potential application has been identified by the recent demonstration of elevated levels of these microRNAs in the cerebrospinal fluid of patients with intracranial malignant GCTs (Terashima *et al*., 2013). Possible applications therefore include diagnosis of malignant GCTs in relatively inaccessible sites, such as the mediastinum, retroperitoneum or central nervous system, without the need for surgery, disease monitoring during chemotherapy and detection of subclinical tumour recurrence which may reduce the need for serial surveillance CT imaging and its inherent secondary cancer risks (Murray & Coleman, 2012). Together, the accumulating data strongly indicate that prospective studies of serum microRNAs in larger patient cohorts are warranted (Murray & Coleman, 2012).

### Under-expressed tissue microRNAs

**Figure 4:**
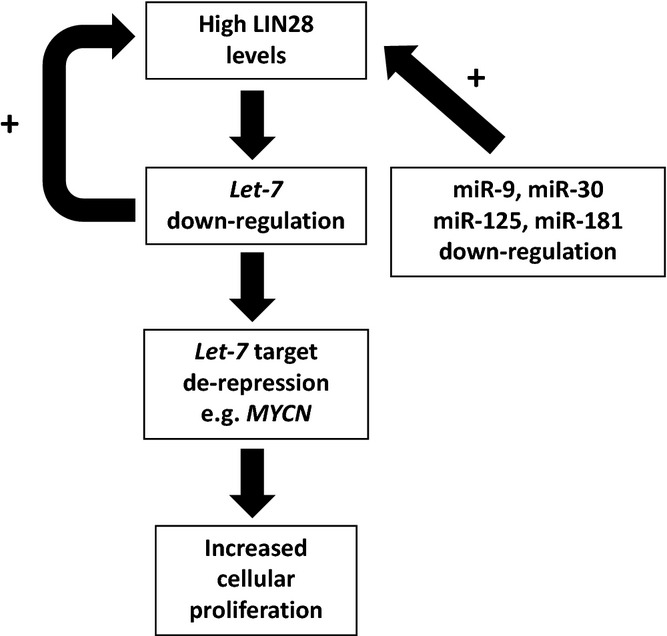
Schematic of the dysregulated *LIN28*/*let-7* axis in malignant germ cell tumours (GCT)s. *Let-7* down-regulation in malignant GCTs is attributable to abundant LIN28 expression (Murray *et al*., 2013). In turn, this leads to over-expression of *let-7* targets (e.g. *MYCN*) and increased cellular proliferation. Loss of direct negative feedback from *let-7* maintains high LIN28 levels and *let-7* repression. Other contributions to abundant LIN28 expression include other microRNAs that bind to the 3′UTR of *LIN28* transcripts, but which are down-regulated in malignant GCTs (Palmer *et al*., 2010). Taken together, these findings highlight the *LIN28*/*let-7* axis as a novel therapeutic target in malignant GCTs.

Members of the *lethal-7* (*let-7*) family of microRNAs, which regulate cell proliferation (Johnson *et al*., 2007) and are important tumour suppressor genes (Takamizawa *et al*., 2004), were under-expressed in paediatric malignant GCTs when compared with non-malignant control tissues (Palmer *et al*., 2010). These microRNAs are specifically negatively regulated by the RNA binding protein *LIN28* (Viswanathan *et al*., 2008, 2009), which is expressed at high levels in PGCs (West *et al*., 2009). Early studies used immunohistochemistry (Cao *et al*., 2011a,b; Xue *et al*., 2011) and RNA interference (Gillis *et al*., 2011) to investigate the expression and some aspects of *LIN28* function in malignant GCTs. For example, *LIN28* depletion in malignant GCT cells led to down-regulation of stem cell markers such as *OCT4/POU5F1* and *NANOG*, and induction of cell differentiation (Gillis *et al*., 2011). However, *LIN28* effects on *let-7* expression were not assessed in this work (Gillis *et al*., 2011). Subsequently, it was shown that the low levels of *let-7* observed in malignant GCTs were directly attributable to abundant *LIN28* expression (Murray *et al*., 2013). As for miR-371–373 and miR-302/367 over-expression, *let-7* under-expression in malignant GCTs was universal, occurring regardless of patient age, histological subtype or site of disease, thereby extending published reports describing predominantly or exclusively tumours from adult patients (Cao *et al*., 2011a,b; Gillis *et al*., 2011; Xue *et al*., 2011). Interestingly, all *let-7* family members contained an identical 2–7nt seed region and the reduced abundance of this seed in malignant GCTs contributed to the subsequent up-regulation of important cancer-associated protein-coding genes, including *MYCN* (Murray *et al*., 2013) (Fig.4). Such post-transcriptional effects on *MYCN* levels are likely to explain why *MYCN* is frequently over-expressed in malignant GCTs (Alagaratnam *et al*., 2011) but shows copy number gain at the *MYCN* 2p23.4 locus in only one third of malignant GCTs from adult patients (Kraggerud *et al*., 2002) and less than one fifth of paediatric cases (Palmer *et al*., 2007).

In addition, low *let-7* levels in malignant GCT cells resulted in increased *LIN28* expression, through a binding site in the *LIN28* 3′UTR for the common *let-7* seed region (Murray *et al*., 2013) (Fig.4). Other microRNAs, including miR-9 (Zhong *et al*., 2010), the miR-30 family (Zhong *et al*., 2010), miR-125 (Wu & Belasco, 2005; Zhong *et al*., 2010) and miR-181 (Li *et al*., 2012) have all been reported to down-regulate *LIN28* in ESCs and cancer cells. Importantly, these microRNAs have previously been identified as being universally under-expressed in malignant GCTs (Murray *et al*., 2013). As copy number gain at the *LIN28* locus (1p36.11) is not a feature of malignant GCTs (Palmer *et al*., 2007), down-regulation of these microRNAs is likely to be an important further contributor to *LIN28* over-expression (Murray *et al*., 2013) (Fig.4). Interactions disrupting this *LIN28*/*let-7* axis represent promising targets for novel therapies in malignant GCTs. As well as depleting *LIN28*, an alternative strategy is direct replacement of *let-7* family members (Murray *et al*., 2013). Both approaches would provide a molecular ‘switch’ effect that should result in a sustained reversion of malignant GCT cell phenotype (Murray *et al*., 2013).

## Conclusion

Our increasing knowledge of the biology of malignant GCTs needs to be harnessed to improve outcomes for both adult and paediatric patients. In particular, genomic and protein-coding transcriptomic data confirm that malignant GCTs of childhood are biologically distinct from those of adulthood and provide evidence supporting the different management approaches employed in patients of different ages. In contrast, all malignant GCTs over-express the miR-371–373 and miR-302/367 clusters regardless of patient age, histological subtype or anatomical tumour site. The detection of elevated serum levels of these microRNAs at the time of malignant GCT diagnosis, with levels falling after treatment, highlights the universal diagnostic potential of this finding. In addition, the dysregulation of the *LIN28*/*let-7* axis in all malignant GCTs also suggests a pathway that may be a target for the development of novel therapeutic agents.

Further studies are now required, integrating genomic changes using high-resolution methods with combined analysis of mRNA and microRNA expression profiles in malignant GCTs from both adult and paediatric patients with good and adverse clinical outcomes. These approaches are likely to identify regulatory pathways and networks associated with treatment sensitivity and resistance. In particular, such studies should aim to define further the clinical relevance of age-specific biological characteristics of malignant GCTs. These insights may assist the optimal management of affected patients and identify biological targets suitable for the development of novel therapeutic agents. The ultimate aim of this approach will be to improve clinical outcomes for this under-investigated patient group, both through improved survival of patients with poor-risk disease and reduced late-effects of treatment for those with low-risk disease.
